# Comparison of Different Physical Therapies Combined with Acupuncture for Poststroke Cognitive Impairment: a Network Meta-Analysis

**DOI:** 10.1155/2021/1101101

**Published:** 2021-11-18

**Authors:** Ruo-Yang Li, Rui-Jue Huang, Qian Yu

**Affiliations:** ^1^Hospital of Chengdu University of Traditional Chinese Medicine, Chengdu, Sichuan 610032, China; ^2^Department of Rehabilitation Medicine, Sichuan Academy of Medical Sciences, Sichuan Provincial People's Hospital, Chengdu, Sichuan 610072, China; ^3^Basic Medical School, Yunnan University of Traditional Chinese Medicine, Kunming, Yunnan 650500, China

## Abstract

**Objective:**

Physical therapy combined with acupuncture is the current research hotspot in the treatment of poststroke cognitive impairment, but which combination treatment is the best is still controversial. Based on the network meta-analysis method, we evaluated the efficacy of various physical therapies combined with acupuncture for the treatment of poststroke cognitive impairment.

**Methods:**

We retrieved diverse randomized controlled trials of various physical therapies combined with acupuncture for the treatment of cognitive dysfunction after stroke. We selected studies, extracted data, and evaluated the risk of literature bias for the included randomized controlled trials. We used STATA 14.0 for the current network meta-analysis.

**Results:**

Fifteen randomized controlled trials involving 1288 patients were included, which involved 7 treatment plans that included 3 control treatment plans and 4 acupuncture treatment plans combined with physical therapy. The best treatment plan for improving the Mini-Mental State Examination score of poststroke cognitive impairment is acupuncture combined with hyperbaric oxygen therapy. The best treatment option for improving the Montreal Cognitive Assessment score of poststroke cognitive impairment is acupuncture combined with hyperbaric oxygen therapy. The best option for improving the Barthel index score of poststroke cognitive impairment is acupuncture combined with transcranial magnetic stimulation. In terms of improving the overall clinical effectiveness of poststroke cognitive impairment, the best treatment option is acupuncture combined with transcranial magnetic stimulation.

**Conclusion:**

The analysis of all the results shows that acupuncture combined with hyperbaric oxygen therapy can significantly improve poststroke cognitive impairment compared with other combined treatments. However, due to the overall quality and quantity of the included studies, more randomized controlled trials focusing on clinical research on acupuncture combined with physical therapy for poststroke cognitive impairment are required to support the current evidence. This trial is registered with CRD42020200092.

## 1. Introduction

Stroke is a global problem that occurs primarily in middle-aged and elderly people. The mortality rate is around 30%. Stroke is usually caused by abnormal cerebral microcirculation due to sudden rupture or blockage of cerebral blood vessels [1, 2]. It has a substantial social and financial burden, with high mortality and morbidity [3]. Some patients who suffer from severe stroke have limb dyskinesias and speech disorders, which leads to a decrease in the quality of their daily life, reduces their contact with the outside world, and reduces their cognitive function [4]. The abovementioned factors induce poststroke cognitive impairment (PSCI). The mortality rate of stroke patients has gradually decreased with the rapid development of medical technology; however, cognitive impairment after stroke is still the major problem that affects the life and work of patients after recovery.

To date, there is no clinically recognized therapy to cure PSCI. Acupuncture is a traditional Chinese medical method whose curative effect has been widely recognized, and many clinical studies have verified that it can effectively ameliorate cognitive dysfunction after stroke. In many countries, especially in Eastern countries, acupuncture has been the first choice for PSCI. In addition to acupuncture, some of the common physical therapies that have been proven to have a significant effect in the treatment of PCSI are transcranial magnetic stimulation (TMS), hyperbaric oxygen therapy (HBO), and ultrashort-wave electrotherapy (UWE). At present, there have been many meta-analyses of acupuncture or physiotherapy intervention on poststroke cognitive function, and the results show its excellent clinical effectiveness [5–8]. In the clinic, to pursue a better curative effect, the combination of acupuncture and physical therapy has become a hot topic in the field of stroke. Clinical studies have proved that acupuncture combined with physical therapy is more effective than single therapy such as pure acupuncture or pure physical therapy in improving cognitive function [9]. A combination of physical therapy and acupuncture can be very effective, but the advantages and disadvantages of their combined effects are not known. Compared with traditional meta-analysis, network meta-analysis can be used to compare multiple interventions, even without direct comparisons.

In this study, a network meta-analysis method was used to evaluate the effect of acupuncture combined with physical therapy for the treatment of PSCI using the outcome indicators of the Mini-Mental State Examination (MMSE), Montreal Cognitive Assessment (MoCA), Barthel index (BI), and the total effective rate of clinical treatment.

## 2. Materials and Methods

### 2.1. Registration

This meta-analysis has been registered on PROSPERO, registration number CRD42020200092.

### 2.2. Literature Search

We searched various databases, including PubMed, EMBASE, the Cochrane Library, APA PsycInfo, CNKI, and WANFANG.

The search strategy used a combination of subject terms and free words. The search time ranged from the establishment of the database to September 2021. The database searches were combined with hand-searching for references reported in the literature. This study focused on the meta-analysis of acupuncture and moxibustion treatment. Taking PubMed as an example, the specific search strategy was as follows:

  (1) acupuncture [Title/Abstract] OR Pharmacopuncture [Title/Abstract] OR “Acupuncture”[Mesh]
  (2) Stroke [Mesh] OR Strokes [Title/Abstract] OR Cerebrovascular Accident [Title/Abstract] OR Cerebrovascular Accidents [Title/Abstract] OR CVAs (Cerebrovascular Accident) [Title/Abstract] OR Cerebrovascular Apoplexy [Title/Abstract] OR Apoplexy, Cerebrovascular [Title/Abstract] OR Vascular Accident, Brain [Title/Abstract] OR Cerebrovascular Stroke [Title/Abstract] OR Cerebrovascular Strokes [Title/Abstract] OR Stroke, Cerebrovascular [Title/Abstract] OR Strokes,
  (3) Cognitions [Title/Abstract] OR Cognitive Function [Title/Abstract] OR Cognitive Functions [Title/Abstract] OR Function, Cognitive [Title/Abstract] OR Functions, Cognitive [Title/Abstract] OR Cognition [Mesh]
  (4) (clinical[tiab] AND trial[tiab]) OR “clinical trials as topic”[mesh] OR “clinical trial”[pt] OR random∗[tiab] OR “random allocation”[mesh] OR “therapeutic use”[sh]
  #1 AND #2 AND #3 AND #4

See Appendix for specific search strategies.

### 2.3. Study Selection

The inclusion criteria were as follows: (1) a randomized controlled trial (RCT); (2) the treatment group was acupuncture combined with physical therapy. Physical therapy refers to the use of physical factors such as sound, light, cold, heat, electricity and force (exercise and pressure) to restore the original state of the body by noninvasive and nondrug treatment for local or systemic dysfunction or lesions of the human body. Other design features, such as blinding, distribution, and allocation concealment were unlimited; (3) the control group was conventional Western medicine treatment or physical therapy by itself; (4) clinical definition of stroke: the stroke diagnosis was confirmed by computed tomography or magnetic resonance imaging; subarachnoid hemorrhage and subdural edema are not included; and (5) the outcome indicator: MMSE, MoCA, BI, and the total effective rate of clinical treatment. When the index is a continuous variable, the included index is the difference before and after treatment, that is, the difference before and after treatment. If it is not mentioned in the original text, it can be calculated from the given data. The formula is as follows, where corr is usually 0.5:

(1) $$\begin{array}{c} {\text{SD}}_{\text{E. change}}=\sqrt{{\text{SD}}_{\text{E. baseline}}^{2}+{\text{SD}}_{\text{E. final}}^{2}-\left(2\times \text{Corr}\times {\text{SD}}_{\text{E. baseline}}\times {\text{SD}}_{\text{E. final}}\right)},\\ {\text{Mean}}_{\text{E. change}}={\text{Mean}}_{\text{E. final}}-{\text{Mean}}_{\text{E. baseline}}. \end{array}$$

Trials were excluded if they met any of the following criteria: (1) the research subjects were nonclinical patients, (2) they were repeated publications, or (3) they had unclear diagnostic criteria or unclear related outcome indicators.

### 2.4. Methodologic Quality

The risk of bias table recommended in Cochrane Handbook 5.1.0 was used to evaluate the quality of the included literature, including reporting bias, detection bias, performance bias, selection bias, attrition bias, and other bias. Each part of the evaluation content can be judged as high, medium, and low risk based on the standards in the Cochrane Handbook 5.1.0.

### 2.5. Data Extraction

Two reviewers conducted a comprehensive and independent review of these publications and cross-checked them. In cases of disagreement, a third party arbitrated. Data items extracted were title, name of the first author, baseline characteristics of the included study (sample size, patient age, treatment measures, etc.), publication year, risk-related factors of bias (randomization method, blinding, etc.), and outcome data (MMSE, MoCA, BI, and total effectiveness). If the above data were unclear, we contacted the corresponding author of the original text.

### 2.6. Statistical Analysis

The statistical method of a network meta-analysis is based on a frequency framework, and all the outcome indicators use the random-effects model for data analysis [10]. If the evaluation indicators of this study were continuous variables MMSE and MoCA, the mean difference (MD) was used as the effect size. When it was a continuous variable outcome indicator BI, then considering that some of the literature measurement methods were improved, the standardized mean difference (SMD) was used. If it was a binary variable, the odds ratio (OR) was used as the effect size, and the corresponding 95% credibility interval (CI) was calculated. STATA 14.0 (Stata Corporation, Lakeway, Texas, USA) was used to draw network evidence relationship diagrams, forest diagrams, grade probability diagrams, funnel diagrams, and the corresponding statistics [10]. When testing global consistency, if the difference was not statistically significant (*P* > 0.05), this indicated that there was no overall inconsistency [11]. This study evaluated local inconsistencies by calculating the inconsistency factors (IFs) and 95% CIs of each closed loop in the network. If the lower limit of the 95% confidence interval contained or was close to 0, the direct comparison evidence was very consistent with the indirect comparison evidence. We calculated the surface under the cumulative ranking curve (SUCRA) probabilities to rank the treatment methods for PSCI. Higher SUCRA values mean better results for the treatment method.

### 2.7. GRADE

Researchers applied the method recommended by the GRADE working group to evaluate the evidence quality of direct and indirect evidence of MMSE in this study [12]. Direct evidence and indirect evidence will be evaluated according to the five aspects bias risk, inconsistency, indirectness, imprecision, and publication bias; and the evidence classification criteria are “very low,” “low,” “moderate,” and “high.” The higher the rating, the closer the estimated effect value to the real effect value.

## 3. Results

### 3.1. Characteristics of the Included Trials

**Figure 1:**
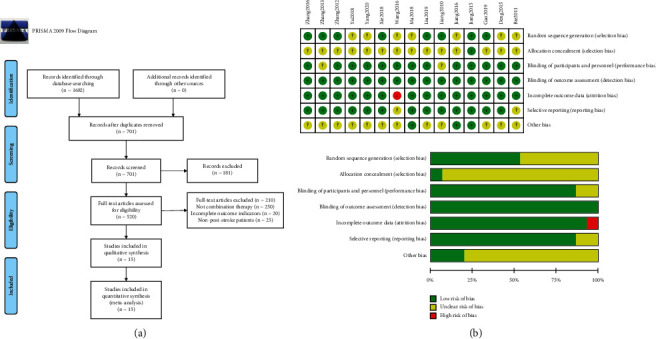
(a) Flow chart of literature screening. (b) Risk of bias graph.

The 15 included trials [13–27] were all conducted in China and included a total of 1288 patients. Four of them were three-armed experiments, and the others were two-armed experiments. The included trials involved 4 types of acupuncture and physiotherapy programs, namely, acupuncture combined with TMS (AT), acupuncture combined with HBO (AH), acupuncture combined with UWE (AU), and acupuncture combined with RehaCom (AR). The included trials had 3 kinds of control treatment plans, namely, conventional treatment (C), acupuncture (A), and HBO (H). The literature screening process and results are shown in Figure 1(a), and the basic information of the included literature is shown in Table 1.

### 3.2. Risk of Bias

The included studies were evaluated by using the Cochrane Manual 5.1.0 bias risk assessment tool. All of the included studies mentioned random allocation, one of them [13] described the random number envelope method as its allocation method, and 5 of them [17, 20, 23–25] described the random number table method as their allocation method. One study [16] described a simple random method as its allocation method, and one study [13] mentioned the single-blind method. The rest of the research is not clearly described. There is one study [19] with no pretreatment data about outcome indicators, while the others have complete outcome data (Figure 1(b)).

### 3.3. Evidence Network

**Figure 2:**
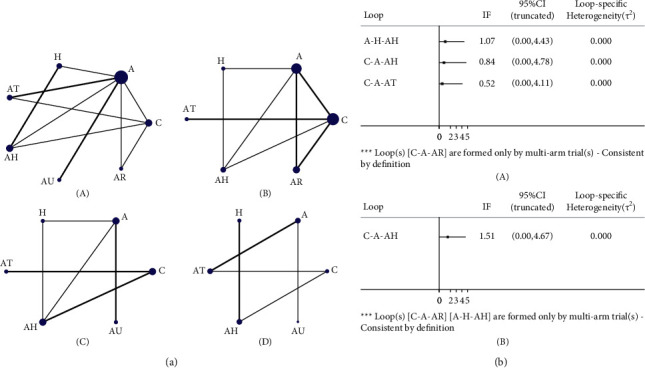
(a) Network diagram (Aa: MMSE; Ab: MoCA; Ac: BI; Ad: total effective rate). (b) Inconsistency (Ba: MMSE; Bb: MoCA).

A total of 9 studies reported MMSE, involving 7 treatment options; a total of 6 studies reported MoCA, involving 6 treatment options; a total of 7 studies reported BI, involving 6 treatment options; and a total of 7 studies reported the total effective rate, involving 6 treatment options. Connecting lines [28] show a direct comparison between 2 connected interventions, and the 2 interventions without a connection can be compared indirectly through a network meta-analysis. The thickness of the lines indicates the number of included studies. The size of the nodes represents the sample size of the included cases using the treatment option. The network diagram is shown in Figure 2(a).

### 3.4. Inconsistency Test

The 7 treatment options of MMSE formed 3 triangular loops. The overall inconsistency test results showed that *P* = 0.8858 (>0.05), indicating that there was no overall inconsistency. The inconsistency factor (IF) was 1.07 (A-H-AH), 0.84 (C-A-AH), and 0.52 (C-A-AT). Their 95% CI of IF reached zero, indicating that they did not have statistical inconsistency. The 6 treatment options of MoCA formed one triangular loop. The overall inconsistency test results showed that *P* = 0.3811 > 0.05, indicating that there was no overall inconsistency. The IF was 1.51 (C-A-AH). The 95% CI of IF reached zero, indicating that no statistical inconsistency existed. The other indicators (BI and total effective rate) did not form a triangular loop, so they did not require the inconsistency test. The results are shown in Figure 2(b).

## 4. Network Meta-Analysis

### 4.1. MMSE

**Figure 3:**
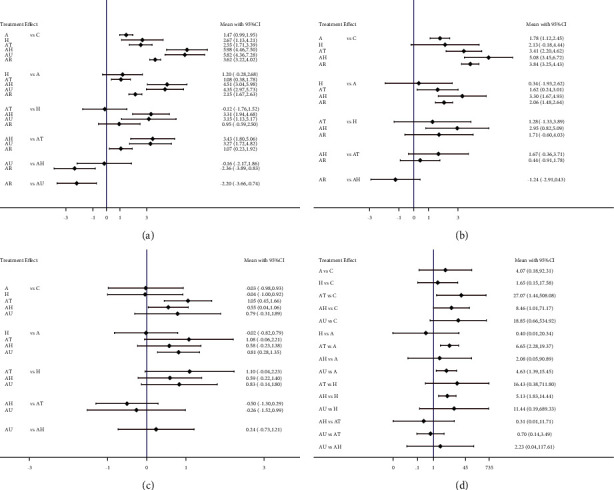
Network meta-analysis: (a) MMSE; (b) MoCA; (c) BI; and (d) the total effective rate.

A total of 7 treatments were compared directly and indirectly, and 17 treatment comparisons were significantly different. Compared with conventional treatment, single acupuncture treatment (MD = 1.47, 95% CI [0.99, 1.95]), HBO treatment (MD = 2.67, 95% CI [1.13, 4.21]), acupuncture combined with TMS (MD = 2.55, 95% CI [1.71, 3.39]), acupuncture combined with HBO (MD = 5.98, 95% CI [4.46, 7.5]), acupuncture combined with UWE (MD = 5.82, 95% CI [4.36, 7.28]), and acupuncture combined with RehaCom (MD = 3.62, 95% CI [3.22, 4.02]) could significantly increase the MMSE score. Acupuncture combined with TMS (MD = 1.08, 95% CI [0.38, 1.78]), acupuncture combined with HBO (MD = 4.51, 95% CI [3.04, 5.98]), acupuncture combined with UWE (MD = 4.35, 95% CI [2.97, 5.73]), and acupuncture combined with RehaCom (MD = 2.15, 95% CI [1.67, 2.63]) could significantly increase the MMSE score and have better curative effects than acupuncture treatment alone. Acupuncture combined with HBO (MD = 3.31, 95% CI [1.94, 4.68]) and acupuncture combined with UWE (MD = 3.15, 95% CI [1.13, 5.17]) could significantly increase the MMSE score and have a good effect compared with HBO. Acupuncture combined with HBO (MD = 3.43, 95% CI [1.80, 5.06]), acupuncture combined with UWE (MD = 3.27, 95% CI [1.72, 4.82]), and acupuncture combined with RehaCom (MD = 1.07, 95% CI [0.23, 1.92]) significantly increased the MMSE score compared with acupuncture combined with TMS. Acupuncture combined with RehaCom (MD = −0.16, 95% CI [−2.17, 1.86]) significantly increased the MMSE score and had a significant treatment effect compared with acupuncture and HBO and acupuncture combined with HBO. Acupuncture combined with UWE significantly increased the MMSE score compared with acupuncture combined with RehaCom (MD = −2.2, 95% CI [−3.66, −0.74]). There was no significant difference for the other treatment comparisons (Figure 3(a)).

### 4.2. MoCA

A total of 6 treatments were compared directly and indirectly, and 8 treatment comparisons had significant differences. Single acupuncture treatment (MD = 1.78, 95% CI [1.12, 2.45]), acupuncture combined with TMS (MD = 3.41, 95% CI [2.20, 4.62]), acupuncture combined with HBO (MD = 5.08, 95% CI [3.45, 6.72]), and acupuncture combined with RehaCom (MD = 3.84, 95% CI [3.25, 4.43]) significantly increased the MoCA score compared with conventional treatment. Compared with single acupuncture treatment, acupuncture combined with TMS (MD = 1.62, 95% CI [0.24, 3.01]), acupuncture combined with HBO (MD = 3.3, 95% CI [1.67, 4.93]), and acupuncture combined with RehaCom (MD = 2.06, 95% CI [1.48, 2.64]) significantly increased the MoCA score. Acupuncture combined with HBO (MD = 2.95, 95% CI [0.82, 5.09]) could significantly increase the MoCA score, and it had a better treatment effect than HBO. There was no significant difference for the other treatment comparisons (Figure 3(b)).

### 4.3. BI

A total of 6 treatments were compared directly and indirectly, and 3 comparisons were significantly different. Acupuncture combined with TMS (SMD = 1.05, 95% CI [0.45, 1.66]) and acupuncture combined with HBO (SMD = 0.55, 95% CI [0.04, 1.06]) could significantly increase the BI score compared with conventional treatment. However, acupuncture combined with UWE (SMD = 0.81, 95% CI [0.28, 1.35]) significantly increased the BI score and had a better curative effect than acupuncture treatment alone. There was no significant difference for the other comparisons (Figure 3(c)).

### 4.4. The Total Effective Rate

A total of 6 treatments were compared directly and indirectly, and 4 comparisons were significantly different. Acupuncture combined with TMS (OR = 27.07, 95% CI [1.44, 508.08]) can improve the total clinical effectiveness compared with conventional treatment. Compared with single acupuncture treatment, acupuncture combined with TMS (OR = 6.65, 95% CI [2.28, 19.37]) and acupuncture combined with UWE (OR = 4.63, 95% CI [1.39, 15.45]) significantly improved the total clinical effectiveness. Compared with HBO, acupuncture combined with HBO (OR = 5.13, 95% CI [1.83, 14.44]) had significantly increased clinical effectiveness; however, no significant difference was observed for the other treatment comparisons (Figure 3(d)).

### 4.5. SUCRA

**Figure 4:**
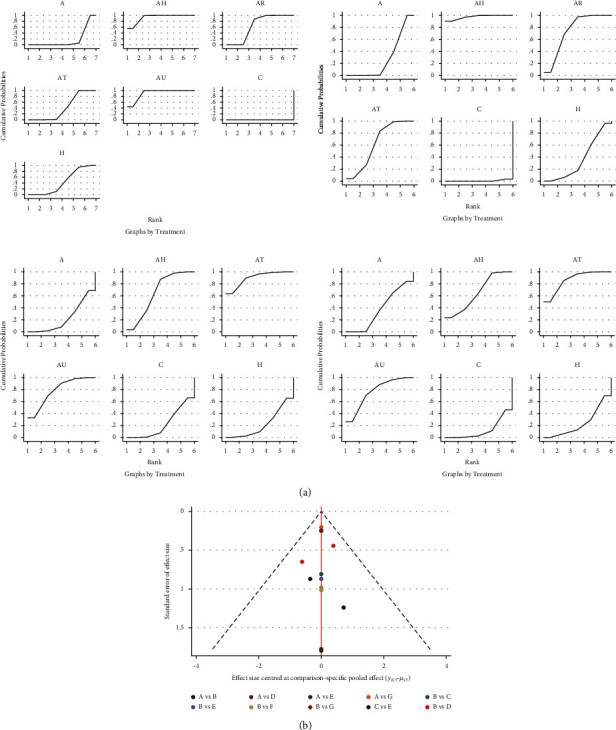
(a) Cumulative probability ranking plot (Aa: MMSE; Ab: MoCA; Ac: BI; Ad: total effective rate). (b) Funnel diagram of MMSE (A: conventional treatment; B: acupuncture; C: HBO; D: acupuncture + TMS; E: acupuncture + HBO; F: acupuncture + UWE; G: acupuncture + RehaCom).

According to the results of SUCRA, acupuncture combined with HBO may be the most effective intervention to increase the MMSE scores. The SUCRA results were as follows: AH (92.6%) > AU (90.7%) > AR (64.7%) > H (43.7%) > AT (40.8%) > A (17.6%) > C (0%). Acupuncture combined with HBO may be the most effective intervention to increase the MoCA score (AH (97.5%) > AR (74.4%) > AT (62.9%) > H (36.6%) > A (27.9%) > C (0.7%)). Acupuncture combined with TMS may be the most effective intervention to increase the BI score (AT (89.7%) > AU (78.1%) > AH (65%) > C (22.7%) > A (22.4%) > H (22.1%)). Acupuncture combined with TMS may be the most effective intervention to increase the total clinical effective rate (AT (86.3%) > AU (76.2%) > AH (64.6%) > A (37.1%) > H (23.7%) > C (12.1%) (Figure 4(a)).

### 4.6. Funnel Chart

In this study, the network meta-analysis method was used. The different colored dots in the funnel diagram of the MMSE score for the PSCI score with various treatment plans indicate direct comparisons between different treatment plans. The number of dots represents the number of studies. Most of the dots in the funnel chart of this study are symmetrically distributed on the vertical line and both sides, and they are symmetrical; however, there may still be a certain degree of publication bias. There were not enough direct comparison experiments between the other indicators, so no bias analysis was performed (Figure 4(b)).

### 4.7. GRADE

In terms of improving the MMSE score of PSCI, 21 statistically significant results were included. Among them, the results of “A vs C,” “AT vs A,” “AU vs A,” and “AH vs H” were rated as moderate quality, the results of “AU vs C,” “AR vs C,” “H vs A,” “AR vs A,” and “AT vs AH” were rated as low quality, and the evidence level of other intervention measures was very low quality. The details are shown in Table 2.

## 5. Discussion

This network meta-analysis involves acupuncture combined with the four most commonly used physical therapy schemes in the clinical treatment of PSCI, namely, acupuncture combined with HBO, acupuncture combined with UWE, acupuncture combined with RehaCom, and acupuncture combined with TMS. Three single treatment plans are involved, namely, common rehabilitation treatment, acupuncture, and HBO.

The results of this study show that the best scheme to improve the PSCI MMSE score is acupuncture combined with HBO followed by acupuncture combined with UWE and acupuncture combined with RehaCom. The best three treatment options for improving the MoCA score of PSCI are acupuncture combined with HBO, acupuncture combined with RehaCom, and acupuncture combined with UWE. MMSE and MoCA are currently the most widely used clinical screening methods for cognitive function. Their scores are correlated with the degree of cognitive dysfunction. A combination of MMSE and MoCA often plays a critical role in the diagnosis and prognosis of PSCI. This study found that acupuncture combined with HBO may be the best combination to improve cognitive function after PSCI. Acupuncture is a core treatment of traditional Chinese medicine, which has a long history, and its applications combined with modern medicine have an objective clinical effect. Western Jin Dynasty's “Zhen Jiu Jia Yi Jin” recorded that acupuncture applied at Lieque (LU7), Tianfu (LU3), and Yongquan (KIl) could treat forgetfulness; acupuncture at Neiguan (PC6) is an effective way to cure mentality; and Shenmen (HT7) masters “sufficiency of spirit and energy.” The “Zhen Jiu Da Cheng” of the Ming Dynasty also documented that “Shenmen (HT7) removes the mind and nature of dementia” and “Baihui (GV20) treats stroke, palpitations, forgetfulness, and trance.” The archives of acupuncture treatment for PSCI have continued throughout the history of traditional Chinese medicine. Previous studies reported [29] that acupuncture can promote learning in rats after cerebral ischemia by increasing the expression of Bcl-2 protein in the hippocampal CA1 area after cerebral infarction, protecting hippocampal neurons from apoptosis, and significantly regulating the number of synapses and the synaptic ultrastructure. There are also many clinical studies demonstrating the effectiveness of acupuncture in treating PSCI. A meta-analysis [30] was conducted to evaluate the effectiveness of acupuncture in treating PSCI, and the results showed that acupuncture was effective in improving PSCI compared with the conventional treatment group, revealing its clinical effectiveness. Because of the excellent curative effect of acupuncture, it has gradually become the routine therapy of PSCI, which is also the reason why acupuncture is selected as the core therapy in this network meta-analysis. It is well known that the mechanism of oxidative stress is closely related to the pathogenesis of cognitive impairment after stroke. HBO increases the amount of dissolved oxygen in the blood, increasing the diffusion radius of blood oxygen, accelerating the formation of collateral circulation, reducing blood flow, inhibiting bacteria, and reducing the intracellular Ca^2+^ concentration. It is widely used in modern medicine [31]. Studies have shown [32] that the use of HBO after stroke can promote the recovery of nerve cells and improve the proliferation and differentiation of endogenous neural stem cells. Combined with the results of this study, acupuncture combined with HBO may be the best choice for patients with a significant decline in cognitive function after stroke.

The best three treatment schemes for the secondary index BI score of this study are acupuncture combined with TMS, acupuncture combined with UWE, and acupuncture combined with HBO, among which acupuncture combined with TMS is the best choice. And the top three best treatment options for improving the overall clinical effectiveness of PSCI are acupuncture combined with TMS, acupuncture combined with UWE, and acupuncture combined with HBO. The BI score is usually used as an auxiliary scoring system to evaluate the quality of daily life of patients. In addition to being related to cognitive function, it is more closely related to the degree of limb movement of patients. Another secondary index the total clinical effective rate is also an evaluation of the comprehensive status of patients with PSCI. Acupuncture and moxibustion not only has a clear effect on improving the cognitive function after stroke but also has been verified clinically and experimentally as an effective measure to treat limb dysfunction after stroke [33]. TMS is widely used in clinical practice due to its advantages of noninvasiveness, effectiveness, safety, and ease of operation. During the last two decades, TMS has been widely investigated to improve blood flow and metabolism in the brain and to affect receptor levels and neuroendocrine function, neuroplasticity, and nerve regeneration [34]. Combined with the results of this study, acupuncture combined with TMS may be the best choice for patients with decreased cognitive function and motor dysfunction after stroke.

While comparing the four outcome indicators included in this study, we found that the optimal ranking of each combination treatment option differs dramatically, so it is difficult to choose the best treatment plan. According to a comprehensive ranking of key indicators, this study found that acupuncture combined with HBO may be the best combination to improve cognitive function after PSCI, whereas acupuncture combined with TMS may be the best intervention to improve the quality of life and achieve overall clinical effectiveness after PSCI. However, compared with these two, acupuncture combined with HBO is more targeted in the treatment of cognitive function.

This study also involves two other treatment schemes, acupuncture combined with UME and acupuncture combined with RehaCom. UWE is a physical therapy method that uses the human body environment to form conduction currents and displacement currents through ultrashort waves and then forms a uniformly distributed shortwave thermal effect, which promotes blood circulation [35]. RehaCom is an effective computer-assisted brain rehabilitation training system evaluated by various investigators. This system consists of a basic program and numerous training items. A previous study confirmed that RehaCom has been widely used in the clinical treatment of PSCI [36]. In this study, although these two combined therapies are not at the top of the list, their ranking is almost superior to single physical therapy or pure acupuncture based on the results of the four indicators. It is suggested that acupuncture combined with physical therapy can usually show better efficacy in the treatment of PSCI than pure acupuncture or single physical therapy.

GRADE rating results show that most of the included interventions are rated as very low quality. The reasons may be as follows: first, most of the included studies are Chinese literature with medium and high risk of bias. Because acupuncture and physiotherapy are difficult to implement blind method, and most Chinese literature does not mention the distribution concealment of patients after enrollment and the blinding of data analysts, the evidence quality of the research results is degraded. The second is that most of the included studies are single-center and small-sample clinical trials, lack of multicenter and large-sample trials, and the evidence accuracy of the research results is low. The third is that the amount of research literature included is small, while the types of intervention measures are complex, the indirect transmission between them is high, and they are small-sample tests. Their position in the probability ranking table is easy to change due to simple factors. The fourth is that the included literature has almost no negative results, resulting in a high possibility of publication bias. Therefore, the overall quality of evidence is not high.

There were several strengths of this network meta-analysis. First, this is the first network meta-analysis to evaluate and compare several different physical therapies combined with acupuncture for the treatment of PSCI. Second, all of the included studies were RCTs, which improved the reliability of the results. Third, we used the SUCRA value to evaluate the subtle differences between treatment plans. Fourth, we used the GRADE scoring system to score the evidence quality of the direct comparison and indirect comparison of the main index MMSE in detail. Nevertheless, this meta-analysis has several potential limitations. First, the number of relevant studies retrieved was too small, especially after applying the inclusion criteria, and all studies were conducted in China, which will lead to great potential selection bias. Second, the included studies did not clearly explain the random allocation method or the allocation concealment. Therefore, selection bias and reporting bias cannot be eliminated, suggesting that the quality of the included studies was not high. Third, the literature rarely mentions adverse reactions. When the outcome indicators of adverse reactions are included, the safety of acupuncture combined with different physical therapies in the treatment of PSCI can be better compared.

## 6. Conclusion

This study preliminarily evaluated the effect of acupuncture combined with physical therapy on PSCI and the improvement of living ability through network meta-analysis and suggested the corresponding best intervention measures, which provided some decision-making evidence for acupuncture combined with physical therapy in the treatment of this disease. According to the results of the network meta-analysis, acupuncture combined with HBO can significantly improve cognitive function after PSCI, while acupuncture combined with TMS can improve the quality of daily life after PSCI compared with other physical therapies. However, due to the overall quality and quantity of included studies, more randomized controlled trials are needed to focus on the effectiveness and safety of acupuncture combined with physical therapy in the treatment of PSCI, so as to provide standardized and effective evidence-based medical evidence for acupuncture combined with physical therapy in the treatment of PSCI.

## Figures and Tables

**Table 1:** Basic characteristics of the included trials.

Author	Year	Number of patients			Age (year)			Male/female	Treatment			Intervention period (wk)	Outcome indicator
		I	C	C2	I	C	C2		I	C	C2		
Jiang [13]	2016	52	49	52	57.88 ± 9.45	56.18 ± 11.86	57.75 ± 13.74	72/81	Acupuncture + RehaCom	Conventional treatment	Acupuncture	12	①, ②
Deng [14]	2015	21	21	—	62.24 ± 10.25			25/17	Acupuncture + TMS	Conventional treatment	—	4	①, ②, ③
Gao [15]	2019	30	30	—	64.33 ± 9.07	58.43 ± 14.02	—	36/24	Acupuncture + TMS	Acupuncture	—	3.7	①, ④
Liang [16]	2010	59	55	—	61.2 ± 10.1	59.2 ± 11.2	—	68/46	Acupuncture + HBO	Conventional treatment	—	3.6	③, ④
Liu [17]	2019	42	41	—	67.6 ± 6.48	68.3 ± 6.75	—	45/38	Acupuncture + UWE	Acupuncture	—	4	①、③
Ma [18]	2018	30	30	30	60.97 ± 7.15	62.32 ± 8.84	60.12 ± 6.56	53/37	Acupuncture + HBO	Acupuncture	HBO	4.3	①
Wang [19]	2016	43	43	—	44.55 ± 30.23	44.45 ± 30.91	—	59/27	Acupuncture + HBO	HBO	—	2.9	④
Xie [20]	2018	45	45	—	63.76 ± 6.84	64.23 ± 6.57	—	50/40	Acupuncture + UWE	Acupuncture	—	4	①, ③, ④
Yang [21]	2020	29	29	28	58.41 ± 8.99	56.55 ± 8.53	60.11 ± 10.05	62/24	Acupuncture + HBO	Acupuncture	HBO	4	②, ③
Yu [22]	2018	30	30	—	63.84 ± 1.55			34/26	Acupuncture + TMS	Conventional treatment	—	2.9	②, ③
Zhang [23]	2012	60	55	—	59.2 ± 8.6	60.2 ± 9.2	—	69/46	Acupuncture + HBO	Conventional treatment	—	2.9	②, ③
Zhang [24]	2013	34	34	—	64.70 ± 8.2	63.10 ± 7.6	—	44/24	Acupuncture + HBO	Conventional treatment	—	2.1	①
Zhang [25]	2016	43	43	—	67.3 ± 5.8	68.2 ± 5.5	—	52/34	Acupuncture + HBO	HBO	—	3	①, ④
Jiang [26]	2015	28	28	27	55.13 ± 7.0	58.20 ± 6.82	56.73 ± 7.648	42/39	Acupuncture + RehaCom	Conventional treatment	Acupuncture	12	②
Bie [27]	2011	36	36	—	61.4 ± 9.8	62.1 ± 10.2	—	38/34	Acupuncture + TMS	Acupuncture	—	4	①, ④

① MMSE; ② MoCA; ③ BI; ④ the total effective rate of clinical treatment; I: Intervention; C: Control

**Table 2:** Effect quantity and evidence classification of MMSE.

Comparative measures	Direct comparison		Indirect comparison		Network meta-analysis	
	MD (95% CI)	Quality of evidence	MD (95% CI)	Quality of evidence	MD (95% CI)	Quality of evidence
A VS C	1.47 (0.92, 2.02)	Moderate^%^	Not evaluated^$^	Not evaluated^$^	1.47 (0.99, 1.95)	Moderate
H VS C	—	—	2.67 (1.13, 4.21)	Very low^&#!^	2.67 (1.13, 4.21)	Very low
AT VS C	3.05 (−0.43, 6.53)	Very low^&%!^	2.52 (1.65, 3.38)	Very low^##!^	2.55 (1.71, 3.39)	Very low
AH VS C	5.45 (1.93, 8.97)	Very low^&%!^	6.10 (4.41, 7.79)	Very low^#∗!^	5.98 (4.46, 7.50)	Very low
AU VS C	—	—	5.82 (4.36, 7.28)	Low^#%^	5.82 (4.36, 7.28)	Low
AR VS C	3.62 (3.14, 4.10)	Low^%!^	Not evaluated^$^	Not evaluated^$^	3.62 (3.22, 4.02)	Low
H VS A	1.11 (−0.47, 2.69)	Low^&!^	1.80 (−2.31, 5.91)	Low^&!^	1.20 (−0.28, 2.68)	Low
AT VS A	1.06 (0.34, 1.78)	Moderate^!^	1.59 (−1.92, 5.10)	Very low^&#!^	1.08 (0.38, 1.78)	Moderate
AH VS A	4.81 (3.11, 6.51)	Very low^&%!^	3.60 (0.60, 6.60)	Very low^&∗!^	4.51 (3.04, 5.98)	Very low
AU VS A	4.35 (2.97, 5.73)	Moderate^!^	Not evaluated^$^	Not evaluated^$^	4.35 (2.97, 5.73)	Moderate
AR VS A	2.15 (1.60, 2.70)	Low^%!^	Not evaluated^$^	Not evaluated^$^	2.15 (1.67, 2.63)	Low
AT VS H	—	—	−0.12 (−1.76, 1.52)	Very low^&#!^	−0.12 (−1.76, 1.52)	Very low
AH VS H	3.36 (1.96, 4.76)	Moderate^%^	Not evaluated^$^	Not evaluated^$^	3.31 (1.94, 4.68)	Moderate
AU VS H	—	—	3.15 (1.13, 5.17)	Very low^&#!^	3.15 (1.13, 5.17)	Very low
AR VS H	—	—	0.95 (−0.59, 2.50)	Very low^&#!^	0.95 (−0.59, 2.50)	Very low
AH VS AT	—	—	3.43 (1.80, 5.06)	Low^&!^	3.43 (1.80, 5.06)	Low
AU VS AT	—	—	3.27 (1.72, 4.82)	Very low^&!∗^	3.27 (1.72, 4.82)	Very low
AR VS AT	—	—	1.07 (0.23, 1.92)	Very low^&!∗^	1.07 (0.23, 1.92)	Very low
AU VS AH	—	—	−0.16 (−2.17, 1.86)	Very low^&!%^	−0.16 (−2.17, 1.86)	Very low
AR VS AH	—	—	−2.36 (−3.89, −0.83)	Very low^&!%^	−2.36 (−3.89, −0.83)	Very low
AR VS AU	—	—	−2.20 (−3.66, −0.74)	Very low^&%!^	−2.20 (−3.66, −0.74)	Very low

&, study limitations; #, indirectness; ∗, inconsistency; %, imprecision; !, publication bias; $, not evaluated, because the treatment protocol is not in the evidence network: indirectness due to nontransitivity or indirectness between the mesh meta-analysis population and the included trial population.

## Data Availability

This paper is a meta-analysis, so all the original data in this paper can be extracted from the cited references mentioned in this paper.

## Appendix

### PubMed

  #1(clinical[tiab] AND trial[tiab]) OR “clinical trials as topic”[mesh] OR “clinical trial”[pt] OR random∗[tiab] OR “random allocation”[mesh] OR “therapeutic use”[sh]
  #2(Stroke [Mesh] OR Strokes [Title/Abstract] OR Cerebrovascular Accident [Title/Abstract] OR Cerebrovascular Accidents [Title/Abstract] OR CVAs (Cerebrovascular Accident) [Title/Abstract] OR Cerebrovascular Apoplexy [Title/Abstract] OR Apoplexy, Cerebrovascular [Title/Abstract] OR Vascular Accident, Brain [Title/Abstract] OR Cerebrovascular Stroke [Title/Abstract] OR Cerebrovascular Strokes [Title/Abstract] OR Stroke, Cerebrovascular [Title/Abstract] OR Strokes)
  #3 Cognitions [Title/Abstract] OR Cognitive Function [Title/Abstract] OR Cognitive Functions [Title/Abstract] OR Function, Cognitive [Title/Abstract] OR Functions, Cognitive [Title/Abstract] OR Cognition [Mesh])
  #4(acupuncture [Title/Abstract] OR Pharmacopuncture [Title/Abstract] OR “Acupuncture”[Mesh])
  #1 AND #2 AND #3 AND #4
  39

### EMBASE

  #5
  33
  #1 AND #2 AND #3 AND #4
  #4
  acupuncture: ab, ti OR “electroacupunctur”: ab, ti OR “electro-acupunctur”: ab, ti OR “acupoint”: ab, ti OR “transcutaneous electric nerve stimulat”: ab, ti OR “percutaneous electrical nerve stimulat”: ab, ti
  #3
  cognition: ab, ti OR “post stroke cognitive impairment”: ab, ti OR “cognitions”: ab, ti OR “cognitive function”: ab, ti OR “cognitive functions”: ab, ti OR “function, cognitive”: ab, ti OR “functions, cognitive”: ab, ti
  #2
  “randomized controlled trial”/exp OR “controlled clinical trial”/exp OR randomized: ti, ab OR placebo: ti, ab OR “drug therapy”: lnk OR randomly: ti, ab OR trial: ti, ab OR groups: ti, ab
  #1
  “cerebrovascular accidents”: ab, ti OR “cva (cerebrovascular accident)”: ab, ti OR “cerebrovascular apoplexy”: ab, ti OR “apoplexy, cerebrovascular”: ab, ti OR “vascular accident, brain”: ab, ti OR “brain vascular accident”: ab, ti OR “brain vascular accidents”: ab, ti OR “vascular accidents, brain”: ab, ti OR “cerebrovascular stroke”: ab, ti OR “cerebrovascular strokes”: ab, ti OR “stroke, cerebrovascular”: ab, ti OR “strokes, cerebrovascular”: ab, ti OR apoplexy: ab, ti OR “cerebral stroke”: ab, ti OR “cerebral strokes”: ab, ti OR “stroke, cerebral”: ab, ti OR “strokes, cerebral”: ab, ti OR “stroke, acute”: ab, ti OR “acute stroke”: ab, ti OR “acute strokes”: ab, ti OR “strokes, acute”: ab, ti OR “cerebrovascular accident, acute”: ab, ti OR “acute cerebrovascular accident”: ab, ti OR “acute cerebrovascular accidents”: ab, ti OR “cerebrovascular accidents, acute”: ab, ti OR “strokes”: ab, ti OR “stroke”: ab, ti OR “cerebrovascular accident”/exp

### Cochrane Library

  #1
  (Cerebrovascular Apoplexy): ti, ab, kw OR (Apoplexy, Cerebrovascular): ti, ab, kw OR (Vascular Accident, Brain): ti, ab, kw OR (Brain Vascular Accident): ti, ab, kw OR (Brain Vascular Accidents): ti, ab, kw OR (Vascular Accidents, Brain): ti, ab, kw OR (Cerebrovascular Stroke): ti, ab, kw OR (Cerebrovascular Strokes): ti, ab, kw OR (Stroke, Cerebrovascular): ti, ab, kw OR (Strokes, Cerebrovascular): ti, ab, kw OR (Acute Cerebrovascular Accident): ti, ab, kw OR (Acute Cerebrovascular Accidents): ti, ab, kw OR (Cerebrovascular Accidents, Acute): ti, ab, kw
  #2
  (Cognition): ti, ab, kw OR (Post stroke cognitive impairment): ti, ab, kw OR (Cognitions): ti, ab, kw OR (Cognitive Function): ti, ab, kw OR (Cognitive Functions): ti, ab, kw OR (Function, Cognitive): ti, ab, kw OR (Functions, Cognitive): ti, ab, kw
  #3
  (Acupuncture): ti, ab, kw OR (electroacupunctur): ti, ab, kw OR (electro-acupunctur): ti, ab, kw OR (acupoint): ti, ab, kw OR (Transcutaneous Electric Nerve Stimulat): ti, ab, kw OR (percutaneous electrical nerve stimulat): ti, ab, kw OR (electroacupunctur): ti, ab, kw OR (electro-acupunctur): ti, ab, kw OR (acupoint): ti, ab, kw OR (Transcutaneous Electric Nerve Stimulat): ti, ab, kw
  #1 AND #2 AND #3
  134

### APA PsycInfo

  S1
  AB Stroke OR AB Strokes OR AB Cerebrovascular Accident OR AB Cerebrovascular Accidents OR AB CVAs (Cerebrovascular Accident) OR AB Cerebrovascular Apoplexy OR AB Apoplexy, Cerebrovascular OR AB Vascular Accident, Brain OR AB Cerebrovascular Stroke OR AB Cerebrovascular Strokes OR AB Stroke, Cerebrovascular
  S2
  MA random allocation OR AB random OR MA therapeutic use OR AB clinical OR AB trial OR MA clinical trials as topic OR AB clinical trial
  S3
  AB Cognitions OR AB Cognitive Function OR AB Cognitive Functions OR AB Function, Cognitive OR AB Functions, Cognitive OR MA Cognition
  S4
  AB acupuncture OR AB Pharmacopuncture OR MA Acupuncture
  S2 AND S3 AND S4 AND S1
  3

### CNKI

  SU =  (“Zu Zhong” + “Nao Que Xue” + “Nao Geng Si” + “Que Xue Xing Zhong Feng” + “Zhong Feng”) AND SU = “Ren Zhi” AND SU  =  (“Zhen Jiu” + “Zhen Ci” + “Dian Zhen” + “Huo Zhen” + “Er Zhen”)
  607

### WANFANG

  Theme: ((“Zu Zhong” or “Nao Que Xue” or “Zu Zhong” or “Nao Zu Zhong” or “Que Xue Xing Zu Zhong” or “Zhong Feng”) and “Ren Zhi” and (“Zhen Jiu” or “Dian Zhen” or “Zhen Ci” or “Huo Zhen” or “Er Zhen”))
  866
